# Psychosocial Oncology: Optimizing Outcomes through Interdisciplinary Care in Head and Neck Oncology

**DOI:** 10.3390/curroncol30070501

**Published:** 2023-07-19

**Authors:** Melissa Henry

**Affiliations:** 1 Lady-Davis Institute for Medical Research, Jewish General Hospital, Montreal, QC H3T 1E2, Canada; melissa.henry@mcgill.ca; 2 Gerald Bronfman Department of Oncology, McGill University, Montreal, QC H4A 3T2, Canada

Head and neck squamous cell carcinomas arise from the mucosal epithelium of the oral cavity (lips, buccal mucosa, anterior tongue, hard palate, floor of mouth, and retromolar trigone), nasopharynx, oropharynx (tonsils, base of tongue, soft palate, uvula, and posterior pharyngeal wall), hypopharynx, and larynx [1]. Globally, head and neck cancers (HNCs) are the seventh most common cancers, with 660,000 new cases and 325,000 deaths yearly. Their incidence is increasing in both developed and developing countries. In high-income countries, the increase is attributable to oropharyngeal cancers linked to human papillomavirus (HPV) infection. Still, it is estimated that tobacco smoking and alcohol misuse are involved behavioral risk factors in 72% [1]. HNC is two to four times more prevalent in men, often diagnosed in an advanced stage, with onset mostly in people aged 50 years and older. Five-year survival is approximately 50% for all cases combined, with hypopharyngeal cancers presenting the worst outcomes [1]. Socioeconomic inequalities have been identified as important drivers of mortality beyond behavioral factors. HPV-positive HNCs are associated with a better prognosis (60% reduction in mortality risk), attributable to younger age, lower comorbidities, and improved anti-tumour immunity and radiosensitivity [1]. Treatment of HNC often includes a combination of surgery, radiation therapy, chemotherapy, and immunotherapy. Disease- and treatment-related burden often involves disfigurement and dysfunction in visible areas involving survival, identity, and the social domain (e.g., eating, speech, breathing, mobility) [1].

The World Health Organization (WHO) “conceptualizes functioning as a dynamic interaction between a person’s health condition, environmental factors, and personal factors” [2]. Psychological and social aspects are considered an integral part of the function and are especially important components to address in oncology. The prevalence of clinical levels of distress is particularly high in head and neck cancer and is likely to compromise a variety of outcomes [3,4,5,6]. Mental health is considered an important source of inequality in access to medical services, with a Lancet Commission calling for integrated care models to address health disparities related to this health dimension [7]. Mood is included in clinical practice guidelines for head and neck cancer and in general. It is also integrated within screening tools for functional outcomes alongside food intake, breathing, speech, and mobility [8]. Collaborative care approaches are also promoted, especially in populations experiencing high levels of distress and treatment burden, as in head and neck cancer. A broader focus, including psychosocial aspects, is key. Psychosocial effects are present all along the cancer trajectory continuum, from the moment of diagnosis through treatments and in survivorship. Treatments for head and neck cancer carry a significant physical toll, implying the need to adjust to a new life, at least temporarily, with a long recovery period post-treatment. The readjustment can be made more complex when patients present a diathesis of previous vulnerabilities, such as concomitant life stressors, psychiatric comorbidities, and social determinants of health, given that they are struggling with additional challenges beyond the already stressful event of a potentially life-threatening disease, its treatments, and associated recovery.

The synergistic combination of novel approaches to conceptualizing physical symptom burden, function, and medical outcomes is leading to advances in the field and new approaches to care. This Special Issue of Current Oncology is designed to showcase research on the psychological and social aspects of head and neck cancer.

Albert et al. examine predictors of body image concerns in a large longitudinal dataset of 224 patients newly diagnosed and treated for head and neck cancer, followed over 12 months. Body image concerns are most intensely experienced immediately post-treatment, with a typical readjustment with time but non-return to pre-treatment levels. The authors propose markers to consider as pre-treatment predictors of body image concerns, including medical variables (i.e., stage, physical function, and disfigurement), psychological distress and denial upon cancer diagnosis, and demographics more susceptible to health disparities (i.e., younger age, female sex, language, and marital status). Unresolved depressive symptoms have an important impact on exacerbating body image disturbances, indicating a need to closely monitor and offer treatment for depression in HNC clinics.

Gascon et al. using a large retrospective observational cohort design, identify a higher risk of cancer-related mortality in patients with HNC reporting self-reported moderate/severe depression on screening tools, as well as higher rates of survival in people participating in routine distress screening. Their study outlines the benefits of screening for distress for patients with HNC using brief screening tools and calls to further study mediators and moderators of these relationships.

Cherba et al. report on a qualitative analysis of video recordings of 88 pre- and post-surgical consultations combined with HNC patient and staff interviews, with a focused analysis on patient–provider conversations around anticipated and actual treatment-related bodily changes. The analyses outline the importance of broader communication integrating patients’ psychosocial adjustment to these changes, rather than a strict focus on survival, cure, and physical recovery with silencing of patients’ concerns. This broader approach can allow patients to feel more prepared for the consequences of their surgeries, as well as lead to a better reintegration into the social realm through physician acknowledgment and validation.

Bobevski et al. validated the Shame and Stigma Scale in 258 patients with head and neck cancer. The scale can be used in both English and French to advance research on issues surrounding shame of appearance, sense of stigma, regret, and speech/social concerns.

Van Beek et al. investigate the association of psychological problems with healthcare use and cost among 558 patients with HNC, using the Netherlands Quality of Life and Biomedical Cohort. They report higher use of health care (primary, supportive, and informal) and healthcare costs in people suffering from symptoms of anxiety, fear of cancer recurrence, and depressive disorder. They also highlight the limited access to psychosocial oncology supports, which can hinder the proper management of distress, suggesting the merit of further study.

Shapiro reviews the literature on the impact of human papillomavirus (HPV) on the global burden of cancer, HPV vaccination as a preventative public health intervention, and the importance of further understanding modifiable and actionable drivers of vaccine acceptance and uptake. She calls for leveraging these factors towards vaccine uptake interventions and improved global PHV vaccine coverage.

Silver et al. review de-escalation strategies in HPV-positive oropharyngeal HNC. They report on novel techniques that reduce long-term morbidities in low-risk patients while maintaining or improving survival rates. Issues around long-term quality of life are especially important to consider as the population of HPV-positive oropharyngeal HNC grows yearly in younger patients with longer survival.

Nehlsen et al. review the potential benefits of and barriers to exercise in survivors of HNC, as well as exercise strategies in this population. They argue for the need for specific guidelines for patients with head and neck cancer, considering their rehabilitative needs post-treatment.

Landry et al. systematically review the literature comparing the health-related quality of life (HrQoL) following total thyroidectomy (TT) versus hemithyroidectomy (HT) in differentiated thyroid carcinoma patients. While they conclude similar psychological and long-term HrQoL, they outline a significant decline in physical and social HrQoL in patients undergoing total versus hemithyroidectomy. These are important considerations in treatment decision-making and consent.

Mascarella et al. present two patients with advanced head and neck cancer struggling with bodily changes. These cases are then discussed by psycho-oncology professionals, opening up a dialogue emphasizing a patient-centered perspective and discussing concrete, evidence-based strategies to facilitate consent, treatment decision-making, and general adaptation.

Dermody et al. review the impact of the pandemic on patients with HNC, outlining how triage decisions and delays in care have had serious psychosocial implications requiring additional structured psychosocial support programs and comprehensive communication.

Together, these articles call for the need for integrated care models to comprehensively address the rehabilitative needs of patients with head and neck cancer, including in the psychological and social domains. Only then can we optimize outcomes and achieve equitable care.

## Data Availability

There is no data associated with this publication.
